# Efficacy and Tolerability of Travoprost 0.004%/Timolol 0.5% Fixed-Dose Combination for the Treatment of Primary Open-Angle Glaucoma or Ocular Hypertension Inadequately Controlled with Beta-Blocker Monotherapy

**DOI:** 10.1155/2017/1917570

**Published:** 2017-01-23

**Authors:** Simon Fabian Lerner, Ki Ho Park, Douglas A. Hubatsch, Valeriy Erichev, Jose A. Paczka, Timothy V. Roberts

**Affiliations:** ^1^Organización Medica de Investigación (OMI), Buenos Aires, Argentina; ^2^Fundación para el Estudio del Glaucoma, Buenos Aires, Argentina; ^3^Seoul National University Hospital, Seoul, Republic of Korea; ^4^Alcon Research, Ltd., Fort Worth, TX, USA; ^5^Research Institute of Eye Diseases, Moscow, Russia; ^6^Instituto de Oftalmologia y Ciencias Visuales, CUCS, Universidad de Guadalajara, Guadalajara, JAL, Mexico; ^7^Unidad de Diagnostico Temprano del Glaucoma, Global Glaucoma Institute Occidente, Guadalajara, JAL, Mexico; ^8^Sydney Medical School, University of Sydney, Sydney, NSW, Australia; ^9^Vision Eye Institute, Sydney, NSW, Australia

## Abstract

*Objective*. To evaluate the efficacy and tolerability of travoprost 0.004%/timolol 0.5% fixed-dose combination (TTFC) in patients with open-angle glaucoma (OAG) or ocular hypertension (OHT) inadequately controlled on beta-blocker monotherapy.* Methods*. In this phase IV, open-label study, 156 patients on beta-blocker monotherapy with mean intraocular pressure (IOP) between 18 and 32 mmHg were randomized (no washout period) to receive TTFC for 8 weeks (TTFC group) or to continue beta-blocker monotherapy for 4 weeks followed by TTFC for the remaining 4 weeks (beta-blocker group).* Results*. The mean IOP (±standard deviation) at baseline in the TTFC and beta-blocker groups was 22.5 ± 2.5 mmHg and 22.2 ± 2.3 mmHg, respectively, and at weeks 4 and 8, was 16.7 ± 3.1 mmHg and 16.1 ± 3.1 mmHg, respectively, in TTFC group and 21.1 ± 3.1 mmHg and 16.1 ± 2.8 mmHg, respectively, in the beta-blocker group. There was a significant least squares mean difference between TTFC and beta-blocker in 8 a.m. IOP at week 4 (−4.6 mmHg; one-sided 95% confidence interval [−inf, −3.9]; *p* < 0.0001 [primary endpoint]); the upper bound of the 95% confidence interval was within the prespecified limit (<0). Both treatments were well tolerated.* Conclusion*. Superior IOP control was achieved with TTFC in patients with OAG or OHT previously uncontrolled with beta-blockers. No new safety findings were identified. This trial is registered with ClinicalTrials.gov NCT02003391.

## 1. Introduction

Elevated intraocular pressure (IOP) is the major and currently only modifiable risk factor for optic nerve damage in open-angle glaucoma (OAG) [1–9]. As a 20% to 30% reduction in IOP from baseline has been shown to substantially reduce the risk of further loss of visual field in OAG patients as well as delay the progression to glaucoma in ocular hypertension (OHT) patients, the aim of treatment is to reduce IOP to a target pressure [6, 8].

Among the currently available treatment modalities, topical hypotensive medications are the preferred initial choice of treatment to lower IOP in glaucoma patients [2, 3]. Of the different classes of topical hypotensives, prostaglandin analogs (PGAs) and beta-blockers are commonly used worldwide as the first line of treatment for OAG and OHT [1, 10, 11].

DuoTrav™ (Alcon Laboratories, Inc., Fort Worth, Texas) is a fixed-dose combination ophthalmic solution containing two topical hypotensives, a PGA travoprost 0.004% and a beta-blocker timolol 0.5% (TTFC). TTFC, through the complementary mechanisms of action of its components, has been shown to lower IOP by 32% to 38% from baseline in patients with OAG or OHT [12–20]. TTFC is approved in Europe, Australia, Latin America, Canada, and several countries in Asia for reducing IOP in adult patients with OAG or OHT who are insufficiently responsive to beta-blockers or PGAs.

A head-to-head comparison of TTFC and beta-blocker treatment in patients with OAG or OHT with insufficient IOP reduction with beta-blocker monotherapy has not been investigated previously. The objective of this phase IV study was to evaluate the efficacy and tolerability of TTFC compared with beta-blocker monotherapy in patients with OAG or OHT who were using a beta-blocker and needed further IOP lowering.

## 2. Methods

### 2.1. Study Design

This 8-week, phase IV, prospective, multicenter, open-label, randomized, parallel-group, postmarketing study was conducted across 14 sites in five countries (Argentina, Mexico, Australia, Russia, and Korea) between December 2013 and May 2015 (Clinicaltrials.gov identifier NCT02003391).

Eligible patients were randomized (1 : 1) to receive either TTFC (1 drop/affected eye, self-administered every evening at approximately 8 p.m.) or continued with their existing beta-blocker monotherapy (1 drop/affected eye twice daily, self-administered at approximately 8 a.m. and 8 p.m.) for the first 4 weeks of the study. From week 5 to week 8, patients in both treatment groups received TTFC (1 drop/affected eye, self-administered at approximately 8 p.m.). There was no washout period between beta-blocker therapy and initiation of TTFC. Patient randomization was stratified by site using a prestudy generated randomization list that was provided to the sites in sealed envelopes. Each patient, upon informed consent, was assigned a 7-digit study number consisting of a 4-digit sponsor-assigned number and a 3-digit sequential screening number. Following verification of eligibility, patients were randomized by sequential assignment of numbers by the designated staff at the investigational site. The proportion of patients from the Latin American and Asian regions were kept close to 1 : 1.

Only one eye from each patient was chosen as the study eye. If both eyes were diagnosed with OAG or OHT and dosed, the worse eye (defined as the eye with higher IOP at baseline) was selected as the study eye. If both eyes were equal at baseline, the right eye was selected as the study eye. The study consisted of three visits: at baseline, week 4, and week 8.

The study was performed in accordance with the principles of the Declaration of Helsinki and in compliance with the Good Clinical Practices. The study protocol was approved by the appropriate independent ethics committee/institutional review board of the participating sites and patients provided signed informed consent.

### 2.2. Patients

Male or female patients, aged ≥18 years, with an existing clinical diagnosis of either OAG (presence of optic nerve and visual field damage compatible with glaucoma and not attributable to any other condition, in addition to open angles in gonioscopy and elevated IOP values between 18 and 32 mmHg) or OHT (open angles in gonioscopy and elevated IOP values and absence of optic nerve and/or visual field damage) were eligible if they were on beta-blocker monotherapy for >30 days, with a mean baseline IOP >18 mmHg and <32 mmHg in at least one eye and in the opinion of the investigator would benefit from further IOP reduction.

Exclusion criteria included any severe central visual field loss (defined as a sensitivity of ≤10 dB in at least 2 of the 4 visual field test points closest to the point of fixation) in either eye within the last year; any chronic, recurrent, or severe inflammatory eye disease (e.g., sclerotic, uveitis, or herpes keratitis); ocular trauma or surgery within the past 6 months; ocular infection or inflammation within the past 3 months; a best-corrected visual acuity (BCVA) score of ≤55 Early Treatment Diabetic Retinopathy Study (ETDRS) letters (equivalent to 20/80 Snellen score, 0.60 logarithm of the minimal angle of resolution [logMAR], or 0.25 decimal); any abnormality that would have prevented reliable measurement of IOP; other ocular pathologies (including severe dry eye) that, in the opinion of the investigator, would have precluded the administration of study medication; conditions that would require the use of any additional topical or systemic ocular hypotensive medication during the study; and hypersensitivity to PGAs or any component of the study medications. Females who were pregnant, those who intended to become pregnant, those not willing to use a highly effective method of birth control during the study period, and those who were lactating were also excluded.

### 2.3. Intervention

The approved formulation of TTFC in Argentina, Australia, and Korea is DuoTrav PQ® (with polyquad as preservative) and in Mexico and Russia is DuoTrav BAK® (with benzalkonium chloride as preservative). Except for the preservative, both formulations are identical in composition. Previous studies have shown that the formulations have similar efficacy and safety profiles [19].

### 2.4. Assessments

IOP measurement, BCVA assessment, and an ocular examination of both eyes were conducted at every visit (baseline, week 4, and week 8).

IOP measurements were performed in both eyes using a Goldmann applanation tonometer with accuracy within ±2 mmHg. The fluorescein and anesthetic agents remained constant throughout the study, and all IOP measurements for any individual subject were preferably performed by the same operator using the same tonometer. IOP measurements were performed at 8 a.m. (±30 min), and two consecutive measurements were recorded for each eye.

Slit lamp examination and BCVA testing preceded IOP measurement. Safety parameters included evaluation of ocular signs (eyelids/conjunctiva, cornea, lens, and iris/anterior chamber) in the right (oculus dextrus [OD]) and left (oculus sinister [OS]) eyes using slit lamp microscopy. BCVA was measured first in the right eye using an ETDRS chart at a distance of 3 meters (10 feet) or 4 meters (13 feet). For patients who could not read English letters, a numerical Tumbling E or a Landolt C logMAR chart was used.

### 2.5. Study Endpoints

The primary endpoint of the study was the difference in 8 a.m. IOP between TTFC and beta-blocker monotherapy at week 4. The secondary endpoints were mean change from baseline and percentage change from baseline in 8 a.m. IOP at week 4. Supportive endpoints included mean change from baseline and percentage change from baseline in 8 a.m. IOP after 4 weeks of TTFC treatment using pooled data (data from baseline to week 4 for the TTFC group and data from week 4 to week 8 for the beta-blocker group were pooled), achievement of IOP ≤18 mmHg at week 4 and week 8, and the 8 a.m. IOP, the mean change from baseline and the percentage change from baseline in 8 a.m. IOP at week 8 with TTFC.

Safety assessments were performed at all study visits and included monitoring and documentation of treatment-emergent adverse events (TEAEs) and assessment of their severity, seriousness, and causal relationship to the treatment; determination of BCVA; and slit lamp examinations of the eyelids, conjunctiva, cornea, iris, and lens.

### 2.6. Statistical Analysis

Assuming a 10% drop-out rate, 156 patients were planned to be randomized (78 per region and 39 per treatment group in each region) to obtain 138 evaluable patients. This sample size had an estimated power of 80% to detect differences between treatments in 8 a.m. IOP at week 4 (IOP in TTFC group lower than IOP in beta-blocker group) if the true difference was >1.5 mmHg between treatments based on the assumption of a standard deviation (SD) of 3.5 mmHg and the use of a two-sample, one-sided *t*-test performed at *α* = 0.05 level of significance.

For the superiority hypothesis, TTFC treatment was considered superior to beta-blocker monotherapy if the least squares means (LSM) difference in the mean 8 a.m. IOPs at week 4 between the treatment groups (TTFC and beta-blocker) was significant (i.e., one-sided *p* value was <0.05, which corresponds to an upper bound of the one-sided 95% confidence interval [CI] of <0). The treatment difference for the primary endpoint was examined using a between-treatment test based on the LSM derived from an analysis of covariance (ANCOVA) model that included region (Latin America [Argentina and Mexico] or Asia [Australia, Russia, and Korea]) and treatment as fixed effects and baseline 8 a.m. IOP as a covariate. No multiplicity adjustments were necessary, as the outcome of the study was based solely on the primary analysis comparing 8 a.m. IOP at week 4 between the two treatment groups. The secondary efficacy endpoints were evaluated using the same ANCOVA model used for the primary analysis. A sensitivity analysis was also performed for the primary and secondary endpoints using the ANCOVA model omitting the region effect. The supportive efficacy endpoints and safety endpoints were summarized descriptively.

All efficacy analyses were based on the intent-to-treat (ITT) population that included all patients who had received study medication and had completed at least one on-therapy study visit. Safety analyses were based on the safety population that included all patients who received the study medication.

## 3. Results

**Figure 1:**
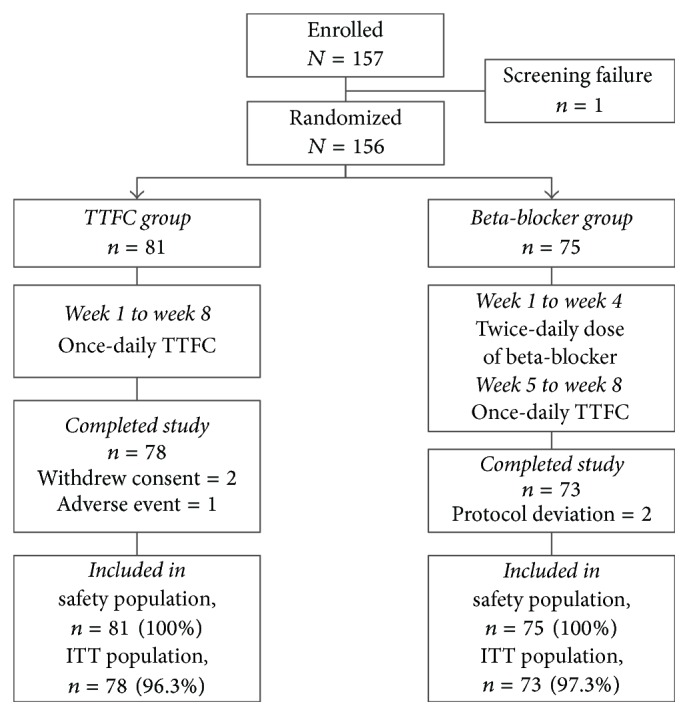
Study design and patient disposition. ITT, intent-to-treat; TTFC, travoprost 0.004%/timolol 0.5% fixed-dose combination.

A total of 157 patients were enrolled and 151 completed the study. One patient was not randomized due to failure to meet the IOP inclusion criteria, and the remaining 156 patients were included in the safety population. The ITT population included 151 patients; of the five excluded patients, two withdrew consent, one withdrew due to an adverse event (AE), and two did not meet the protocol-specified visit window (Figure 1).

The mean age of the study population was 63.3 years (range, 20–86 years); 70.2% of the patients were female, 72.8% were white, and the diagnosis at enrollment was OAG in 78.8% patients. The baseline and demographic characteristics were similar between the TTFC and beta-blocker groups (Table 1).

### 3.1. Efficacy Outcomes

The mean IOP (±SD) in the TTFC and beta-blocker groups was 22.5 (±2.5) mmHg and 22.2 (±2.3) mmHg, respectively, at baseline. At week 4, the mean IOP (±SD) was 16.7 (±3.0) mmHg in the TTFC group and 21.4 (±3.0) mmHg in the beta-blocker group. At week 8, the mean IOP (±SD) was 16.1 (±3.1) mmHg in the TTFC group and 16.1 (±2.8) mmHg in the beta-blocker group.

### 3.2. Primary Outcome

**Figure 2:**
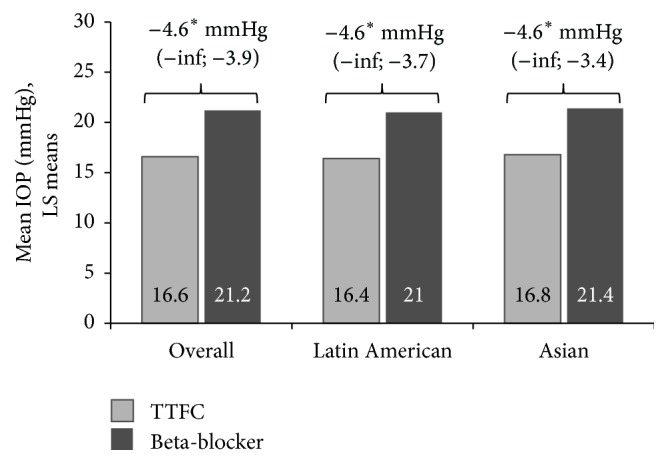
Least squares mean 8 a.m. IOP at week 4 by treatment groups (total population) and by region (ITT population). ^*∗*^ANCOVA results for LS mean difference (one-sided 95% CI) between treatment groups (TTFC and beta-blocker); *p* < 0.0001.* Note*. The *p* values for regions (Latin America and Asia) are for descriptive purposes only. ANCOVA, analysis of covariance; CI, confidence interval; IOP, intraocular pressure; ITT, intent-to-treat; LS, least squares; TTFC, travoprost 0.004%/timolol 0.5% fixed-dose combination.

At week 4, the mean 8 a.m. IOP was lower in the TTFC group (16.7 ± 3.1 mmHg) than in the beta-blocker group (21.2 ± 3.1 mmHg). The LSM difference in 8 a.m. IOP at week 4 between the treatment groups was statistically significant (point estimate: −4.6 mmHg; one-sided 95% CI [−inf, −3.9]; *p* < 0.0001). The superiority of TTFC over beta-blocker monotherapy was established as the upper bound of the one-sided 95% CI was <0 (Figure 2).

The results of the sensitivity analysis stratified by region were similar to those observed for the overall group (Figure 2).

### 3.3. Secondary and Supportive Outcomes

**Figure 3:**
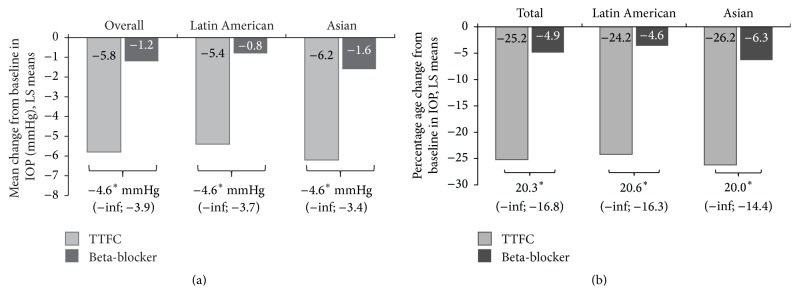
(a) Least squares mean change from baseline^a^ in 8 a.m. IOP at week 4 by treatment groups (total population) and by region (ITT population). ^a^refers to beta-blocker-treated baseline; ^*∗*^ANCOVA results for LS mean difference (one-sided 95% CI) between treatment groups (TTFC and beta-blocker); *p* < 0.0001.* Note*. The *p* values for regions (Latin America and Asia) are for descriptive purposes only. ANCOVA, analysis of covariance; CI, confidence interval; IOP, intraocular pressure; ITT, intent-to-treat; LS, least squares; TTFC, travoprost 0.004%/timolol 0.5% fixed-dose combination. (b) Least squares mean percentage change from baseline^a^ in 8 a.m. IOP at week 4 by treatment groups (total population) and by region (ITT population). ^a^refers to beta-blocker-treated baseline; ^*∗*^ANCOVA results for LS mean difference (one-sided 95% CI) between treatment groups (TTFC and beta-blocker); *p* < 0.0001.* Note*. The *p* values for regions (Latin America and Asia) are for descriptive purposes only. ANCOVA, analysis of covariance; CI, confidence interval; IOP, intraocular pressure; ITT, intent-to-treat; LS, least squares; TTFC, travoprost 0.004%/timolol 0.5% fixed-dose combination.

At week 4, the LSM change and the percentage change from baseline in 8 a.m. IOP were greater in the TTFC group compared with the beta-blocker group (*p* < 0.0001; Figures 3(a) and 3(b)).

**Figure 4:**
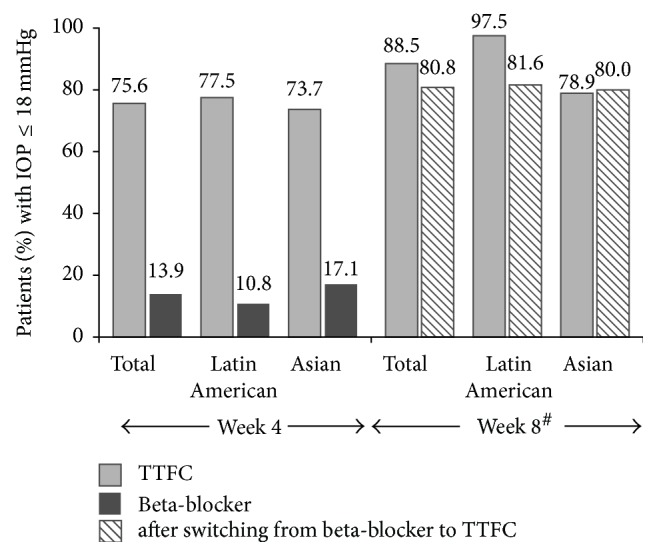
Percentage of patients achieving IOP ≤ 18 mmHg at week 4 and week 8 (total population) and by region (ITT population). ^#^At week 8, both groups were receiving TTFC, as patients in the beta-blocker group had switched to TTFC between week 5 and week 8. IOP, intraocular pressure; ITT, intent-to-treat; TTFC, travoprost 0.004%/timolol 0.5% fixed-dose combination.

Analysis of pooled data also showed a reduction in mean change and percentage change from baseline in 8 a.m. IOP after 4 weeks of treatment with TTFC (mean change: −3.5 ± 3.9 mmHg; percentage change: −15.3% ± 16.6%). As shown in Figure 4, overall, a higher proportion of patients in the TTFC group compared with the beta-blocker group achieved IOP ≤18 mmHg at week 4 (75.6% versus 13.9%). At week 8, the proportion of patients achieving IOP ≤18 mmHg increased in the beta-blocker group following a switch to TTFC after week 4 (Figure 4).

Among patients receiving TTFC treatment for 8 weeks, the mean change in 8 a.m. IOP from baseline was −6.5 ± 3.3 mmHg, and the mean percentage change from baseline in 8 a.m. IOP was −28.3% ± 12.6%.

The exploratory sensitivity analysis stratified by region showed results similar to those of the overall group for the LSM change from baseline and the percentage change from baseline in 8 a.m. IOP at week 4 (Figures 3(a) and 3(b)). Similarly, at week 4, a higher proportion of patients in the TTFC group achieved IOP ≤18 mmHg compared with the beta-blocker group (Latin America: 77.5% versus 10.8%; Asia: 73.7% versus 17.1%). In the TTFC group, at week 8, a higher proportion of Latin American patients achieved IOP ≤18 mmHg compared to Asian patients (97.5% versus 78.9%; Figure 4). The mean change in 8 a.m. IOP from baseline at 8 weeks among patients receiving TTFC treatment was −6.4 ± 2.5 mmHg and −6.5 ± 3.9 mmHg in Latin American and Asian patients, respectively. The corresponding mean percentage change from baseline in 8 a.m. IOP was −29.9% ± 10.57% and −27.5% ± 14.7%, respectively.

### 3.4. Safety Outcomes

Overall, 35 TEAEs were reported during the 8-week study period: 22 TEAEs in 18 patients in the TTFC group and 13 TEAEs in nine patients in the beta-blocker group. As shown in Table 2, the most frequent (≥2%) TEAEs were ocular hyperemia, eye pruritus, dry eye, and nasopharyngitis in the TTFC group and dry eye, nasopharyngitis, and influenza in the beta-blocker group.

Treatment-related TEAEs were reported in 12 patients (14 AEs) in the TTFC group and in three patients (3 AEs) in the beta-blocker group (after switching to TTFC). The most frequently reported treatment-related AEs were ocular hyperemia and eye pruritus in the TTFC group (Table 3). Three patients reported treatment-related AEs in the beta-blocker group, between weeks 5 and 8 of the study, when patients had received TTFC (Table 3).

Two patients in the TTFC group discontinued the study due to treatment-related AEs. One patient withdrew due to ocular hyperemia, 1 day after start of treatment. The other patient withdrew due to moderate eye pruritus and hypersensitivity to treatment, 15 days after start of treatment. No serious adverse events (SAEs) or deaths were reported during the study.

There were no changes from baseline in BCVA at week 4 and week 8 in either treatment group. No clinically relevant changes were observed on slit lamp examination over the 8-week period, except for conjunctival abnormality in approximately 10% of patients in both groups (TTFC, 10.3% OD/10.3% OS; beta-blocker, 9.6% OD/11.0% OS).

## 4. Discussion

This study demonstrated the superior efficacy of once-daily dosing of TTFC in lowering the IOP in patients with OAG and OHT with an insufficient response to existing beta-blocker monotherapy. There was an additional 25% (−5.8 mmHg) mean reduction in IOP levels at week 4 with TTFC as compared with a 4.7% (−1.1 mmHg) mean reduction observed with beta-blocker therapy. The higher IOP lowering with TTFC gains significance considering that this was additional reduction achieved from the beta-blocker-treated baseline (i.e., in treatment-exposed patients) without any washout period between prior and current treatments, mimicking everyday practice. The IOP reduction observed in the beta-blocker group at week 4 may be attributable to the Hawthorne effect, which is often observed in switch studies. Overall, 8-week treatment with TTFC resulted in a net 28% or 6.5 mmHg mean reduction in IOP. These mean reductions in IOP with TTFC are clinically relevant, as a 1 mmHg reduction in IOP is reported to be associated with a 10% lowering in the risk of disease progression in glaucoma patients [9].

Significant IOP control (an additional 5.0 to 5.7 mmHg mean reduction in IOP over a 4- to 16-week period) with TTFC in patients inadequately controlled on beta-blocker monotherapy has been reported in previous observational and open-label, noncomparator studies [15–17, 20]. Similarly, effective improvements in IOP have also been reported with TTFC in patients having an insufficient IOP reduction with PGA monotherapy [15, 16, 21]. Further, studies have shown that TTFC provides greater IOP reduction than its components travoprost and timolol, used separately as single agents [12, 13]. The long-term IOP-lowering efficacy of TTFC has been proven in studies with up to 12 months of follow-up [18, 22].

It has been observed that approximately 40% to 50% of glaucoma patients invariably require more than one medication to achieve their target IOP and therefore have to be switched to a more powerful therapy or need an add-on medication to their existing therapy [2, 6]. At present, treatment with topical hypotensives follows a step-wise approach, wherein if appropriate response to initial monotherapy is not achieved, patients are transitioned to another antiglaucoma agent of the same or different class. PGA and beta-blocker fixed-dose combinations are more effective in reducing IOP compared with timolol monotherapy [23]. A meta-analysis including 40 randomized clinical trials showed that treatment with fixed-dose combinations containing timolol can result in an IOP percentage reduction of >30% from baseline [24]. Fixed-dose combination therapies also provide additional advantages of longer-lasting effect, simple dosing regimen, and better compliance as well as a lower risk of ocular surface disease in patients due to reduced exposure to preservatives, when compared to concomitant administration of their components [25]. Hence, the results from this study and previous studies suggest that transitioning to a fixed-dose combination such as TTFC can be considered to reach target IOP faster, in patients with inadequate response to their existing monotherapy treatment [15–17, 20].

Interestingly, nearly 20% more Latin American patients achieved IOP ≤18 mmHg at week 8 compared with Asian patients. The clinical relevance of this finding is not known, as the mean IOP-lowering efficacy of TTFC was similar in the two regional groups. It should also be noted that the study was not powered to analyze the difference between the two regional groups.

Both treatments were generally well tolerated, with overall low rates of AEs reported in this study. Only two patients withdrew from the study due to ocular AEs in the TTFC group. The most commonly reported drug-related AE with TTFC was hyperemia, which is attributable to the PGA component of the combination. Higher AE rates have generally been reported with fixed combination therapies, likely due to the presence of two components. Consistent with this observation, the incidence of AEs and treatment-related AEs in the study were higher in the TTFC group than in the beta-blocker group. No SAEs or new safety findings were reported with TTFC during the 8-week study period, and the safety profile of TTFC in this study was consistent with that reported in previous studies [12–20].

This phase IV study had both strengths and limitations. The lack of washout period before switching therapies, which ensured that patients received continuous medical care for controlling IOP, is similar to actual clinical practice and adds strength to the present findings. Limitations of the study include the open-label design, lack of long-term follow-up, and a lack of adjustment for multiplicity.

European Glaucoma Society guidelines recommend that aggressive treatment could be required in some glaucoma cases, such as in young patients or those with severe disease, to ensure that the quality of life is sustained by minimizing loss to visual function [1]. The results from this study suggest that patients with glaucoma whose IOP is inadequately controlled with beta-blockers may derive clinical benefit from earlier transition to TTFC.

## 5. Conclusion

Travoprost 0.004%/timolol 0.5% fixed-dose combination was superior to beta-blocker monotherapy in lowering IOP in patients with OAG or OHT inadequately controlled on beta-blocker monotherapy. Both treatments were well tolerated, and no new safety findings were identified.

## Figures and Tables

**Table 1:** Demographics and baseline characteristics (ITT population).

	TTFC (*n* = 78)	Beta-blocker (*n* = 73)	Overall (*N* = 151)
Mean age ± SD (range), years	62.8 ± 12.8 (20–86)	63.8 ± 13.3 (22–86)	63.3 ± 13.0 (20–86)
Gender			
Female, *n* (%)	54 (69.2)	52 (71.2)	106 (70.2)
Race, *n* (%)			
White	58 (74.4)	52 (71.2)	110 (72.8)
Asian	12 (15.4)	10 (13.7)	22 (14.6)
Others	8 (10.3)	11 (15.1)	19 (12.6)
Underlying eye disease, *n* (%)			
OAG	61 (78.2)	58 (79.5)	119 (78.8)
OHT	17 (21.8)	15 (20.5)	32 (21.2)

ITT, intent-to-treat; OAG, open-angle glaucoma; OHT, ocular hypertension; SD, standard deviation; TTFC, travoprost 0.004%/timolol 0.5% fixed-dose combination.

**Table 2:** Treatment-emergent AEs with incidence ≥2% during the study (safety population).

MedDRA preferred term	TTFC (*N* = 81)	Beta-blocker (*N* = 75)
*Total AEs, n (%)*	*18 (22.2)*	*9 (12.0)*
Ocular hyperemia	5 (6.2)	0
Dry eye	2 (2.5)	2 (2.7)
Eye pruritus	4 (4.9)	0 (0.0)
Nasopharyngitis	2 (2.5)	2 (2.7)
Influenza	0	2 (2.7)

A subject with more than one event in a specific category was only counted once. Number of events based on MedDRA coding; due to splitting of terms, number of MedDRA terms can be different from number of reported events. MedDRA version 18.0.

AEs, adverse events; MedDRA, Medical Dictionary for Regulatory Activities; TTFC, travoprost 0.004%/timolol 0.5% fixed-dose combination.

**Table 3:** Incidence of treatment-related TEAEs during the study (safety population).

MedDRA preferred term	TTFC (*N* = 81)	Beta-blocker (*N* = 75)
*Eye disorders, n (%)*	*12 (14.8)*	*3 (4.0)*
Ocular hyperemia	5 (6.2)	0
Ocular pruritus	3 (3.7)	0
Conjunctivitis allergic	1 (1.2)	1 (1.3)
Dry eye	1 (1.2)	1 (1.3)
Ocular surface disease	1 (1.2)	1 (1.3)
Eyelid-pigmentation	1 (1.2)	0
Eye irritation	1 (1.2)	0
Eye pain	1 (1.2)	0
Eyelids pruritus	1 (1.2)	0
*Immune system disorders, n (%)*	*1 (1.2)*	
Hypersensitivity	1 (1.2)	0

A subject with more than one event in a specific category was only counted once. MedDRA coding; due to splitting of terms, number of MedDRA terms can be different from number of reported events. MedDRA version 18.0.

MedDRA, Medical Dictionary for Regulatory Activities; TEAEs, treatment-emergent adverse events; TTFC, travoprost 0.004%/timolol 0.5% fixed-dose combination.
